# Validating Bayesian truth serum in large-scale online human experiments

**DOI:** 10.1371/journal.pone.0177385

**Published:** 2017-05-11

**Authors:** Morgan R. Frank, Manuel Cebrian, Galen Pickard, Iyad Rahwan

**Affiliations:** 1 Media Laboratory, Massachusetts Institute of Technology, Cambridge, MA, United States of America; 2 Data61 Unit, Commonwealth Scientific and Industrial Research Organization, Melbourne, Victoria, Australia; 3 Google Inc., Mountain View, CA, United States of America; National Taiwan University, TAIWAN

## Abstract

Bayesian truth serum (BTS) is an exciting new method for improving honesty and information quality in multiple-choice survey, but, despite the method’s mathematical reliance on large sample sizes, existing literature about BTS only focuses on small experiments. Combined with the prevalence of online survey platforms, such as Amazon’s Mechanical Turk, which facilitate surveys with hundreds or thousands of participants, BTS must be effective in large-scale experiments for BTS to become a readily accepted tool in real-world applications. We demonstrate that BTS quantifiably improves honesty in large-scale online surveys where the “honest” distribution of answers is known in expectation on aggregate. Furthermore, we explore a marketing application where “honest” answers cannot be known, but find that BTS treatment impacts the resulting distributions of answers.

## Introduction

Subjective judgements play an important role in several areas of polling [1, 2] and research [3, 4]. However, subjectivity raises concerns about the accuracy, honesty, and usefulness of responses [5, 6]. For example, political polling is a prominent tool in election prediction, but exit polling can misrepresent the true belief of a population [7, 8]. One plausible explanation from research suggests that response to political polls is swayed by social influence [9] or hidden political agendas [10]. As another example, customer satisfaction is an area relying heavily on subjective response; responders to these surveys may experience pressure from external media or their peers that alter their otherwise true responses [11–13]. In particular, researchers have investigated the effects of social influence on cultural labor markets, and there is evidence that subjective participants in both artificial [14, 15] and real-world markets are not immune to influenced opinions [16–18].

What can cause survey responders to reply dishonestly or apathetically? Social and media pressure are common culprits and their psychological effects can influence spending habits [19], political ideals [20], happiness [21], and the future of social processes [22, 23]. Also, people are profit maximizing [24] by producing responses according to financial gain. It has been shown that people maintain a “self-concept” that allows them to engage in dishonesty without updating their mental model of themselves [25, 26].

Greed, often aided by the shroud of anonymity, is another major confounding variable in survey [27]. Whether internally or externally derived, greed has caused dishonesty in several existing studies covering a variety of test subjects ranging from children [28] to bankers [29]. There exist methods in the literature to combat dishonest profit-maximizing behaviors, such as invoking religion [30, 31], but only recent work has examined quantitative counter-measures rather than emotional ones.

Marketing departments are constantly performing surveys assessing the market value of products or services. However, obtaining thoughtful results from survey participants can be difficult as it is not obvious how to incentivize or measure the effort in their answers while decentivizing greed. Furthermore, improvements in technology and online survey platforms have shifted towards performing these surveys on web-platforms, such as Amazon’s Mechanical Turk, where, typically, participants are paid a pre-determined reward for survey completion. This paradigm fails to incentivize responders to participate thoughtfully, but, instead, to maximize their personal profit by completing multiple surveys as quickly as possible. As a result, this prevalent paradigm breeds apathetic or dishonest survey responses.

Some methods exist for surveyors to assess the apathy of responders, such as assessing survey completion time [32] or inserting trick questions [33]. However, the optimal strategy for responders on web-platforms remains unchanged. Bayesian truth serum (BTS) is a quantitative method for incentivizing truthful responses to subjective multiple-choice survey questions [34]. By design, the effectiveness of BTS increases with the number of responses [35], but, to date, BTS has been shown to combat dishonesty in only small-scale experiments where deceit is explicitly incentivized [36] and to estimate the prevalence of questionable research practices [37]. For BTS to become a readily accepted survey tool, we must demonstrate its effectiveness in surveys at the same scale as real-world applications.

BTS relies on the Bayesian assumption that people maintain a mental model of the world that is biased by their personal experiences, which leads to a belief that personally held opinions are disproportionately present amongst peers. In an effort to understand the effectiveness of BTS, it is essential to test the prevalence of this assumption. If this assumption holds for a variety of scenarios and across populations, then BTS may be a widely applicable method to improve survey response.

In the remainder of this manuscript, we explain the details of the BTS algorithm and describe our specific experimental designs in the Materials and Methods section. The Results section displays the responses to our experiments and assesses the prevalence of the required Bayesian assumption underlying BTS. Finally, we contextualize our findings about the effects of BTS in the Discussion section.

## Materials and methods

### Bayesian truth serum

Bayesian truth serum (BTS) is a method for rewarding honesty or information-gained from responses to subjective multiple-choice questions. Responders are rewarded according to a information score, called “iscore”, and a prediction accuracy score. Let *Q* denote the set of multiple-choice questions comprising a survey; note that the number of options for each question may vary. For multiple-choice question *q* ∈ *Q* with *m* options, we ask responder *i*, for *i* ∈ {1, 2, …, *n*}, to endorse an option which represents their belief and to predict the proportion of responders who will endorse each of the *m* options. An endorsement of option *j*, for *j* ∈ {1, 2, …, *m*}, receives

$$iscore_{q}\left(j\right)=\log\left(\frac{{\bar{x}}_{j}}{{\bar{y}}_{j}}\right),$$ (1)

where ${\bar{x}}_{j}$ is the proportion of the *n* responders endorsing option *j* as their belief, and ${\bar{y}}_{j}$ is the geometric mean of the endorsement predictions for option *j*. Specifically, let

$$I_{j}\left(i\right)=\left\{\begin{array}{cc} 1, & \text{if}\;\text{responder}\;i\;\text{endorses}\;\text{option}\;j\\ 0, & \text{otherwise} \end{array}\right.$$

and let

$$P_{j}\left(i\right)\in \left[0,1\right]\;\text{such}\;\text{that}\;\sum_{j=1}^{m}P_{j}\left(i\right)=1$$

denote responder *i*’s prediction of the proportion of participants endorsing option *j*, then we calculate

$${\bar{y}}_{j}=\exp\left(\frac{1}{n}\sum_{i=1}^{n}\log\left(P_{j}\left(i\right)\right)\right)$$ (2)

and

$${\bar{x}}_{j}=\frac{1}{n}\sum_{i=1}^{n}I_{j}\left(i\right).$$ (3)

Responders are rewarded according to the sum of their iscores and accuracy of predictions across each multiple-choice question in the survey; that is a responder’s score for an individual question is given by

$$score\left(i\right)=iscore_{q}\left(j\right)+\alpha \sum_{j=1}^{m}{\bar{x}}_{j}\log\left(\frac{P_{j}\left(i\right)}{{\bar{x}}_{j}}\right).$$ (4)

This incentive structure leads to honesty being a Bayesian Nash equilibrium [34] for *α* > 0 and is a zero-sum game for *α* = 1. We take *α* = 1 for the experiments described below.

### Experimental design

Our main focus is to demonstrate the effects of BTS treatment in large-scale experiments. The recent advent of several web-survey platforms allows researchers to easily launch surveys and experiments with hundreds or thousands of participants; of these, we use Amazon’s Mechanical Turk (MTurk) because of flexibility in payment options and transparency in responder selection criteria. MTurk provides responders embodying a range of demographics [38, 39], but we restrict our investigation to responders in the United States. Responders from MTurk choosing to participate in our study follow a hyperlink to our survey website and return to MTurk with a unique survey code upon survey completion, which is used to pay out monetary rewards.

For many real-world surveys, the underlying ground-truth is unknown, and, therefore, it can be difficult to validate responses in BTS treatments or the control treatment. Several investigations into survey honesty have overcome this setback by asking responders to privately perform a stochastic task, such flipping a coin [28, 34] or rolling a die [27], while explicitly incentivizing particular responses. The aggregate honesty of responders is seen by comparing the distribution of responses to the expected distribution if the task were performed honestly (i.e. uniform distributions).

In experimental treatment groups subject to BTS incentives, we explain that we will be assigning a score to their selection and predictions which is designed to measure their honesty and accuracy. Responders with iscores ranking them in the top 1/3rd of responders at the completion of the experiment received an additional monetary bonus. We do not reveal how the iscore is calculated in case this knowledge could be exploited. Along with explicitly informing responders that we will reward bonuses according to prediction accuracy, we display the following description of iscores to participants in the BTS treatments:

*Recent work by researchers at MIT has lead to the development of an algorithm for detecting truth telling and information*. *We will assign an* **iscore** *to your response below which indicates how truthful and informative you are being about the average person*. *Once we have collected all of the responses to this survey*, *we will rank the survey responders by the sum of their information scores and award a bonus to the responders in the top 1/3rd*. *This bonus is in addition to the base pay for participating in the survey*.

In our study, we consider two variations of BTS as separate experimental treatments. Responders in the **transparent BTS** treatment will see the dynamically calculated iscores next to each option in a given multiple-choice question, while responders in the **BTS intimidation** treatment do not see the iscores, but are still subject to rewards according to iscores. These two treatments differentiate if any improvement from using BTS in comparison to a control group is due to the actual influence of iscores or from the threat of a truth-detection algorithm.

We perform three experiments in increasing complexity while assessing BTS intimidation and transparent BTS in comparison to a control where no dishonesty counter-measures where undertaken. We perform two experiments involving coin flips and dice rolls where the ground-truth is known in expectation. These experiments allow us to compare the performance of BTS as the spectrum of dishonest choice increases in options. Finally, we use BTS in a realistic pricing survey to demonstrate the real-world applicability of BTS at scale. Our hypothesis is that BTS will improve responses in the experiments where ground-truth is known, and will produce different but sensible responses when the ground-truth can not be known. We provide screen shots of the survey websites in the Section 6 of S1 File.

#### Coin Flip experiment

Responders in the Coin Flip experiment are randomly placed into a BTS intimidation treatment (number of responders: *N* = 1822) or control treatment (*N* = 2032) when the survey webpage loads. Each responder receives a base pay of $0.05 for completing the survey. Responders in the BTS intimidation treatment were informed that an algorithm is in use to assess their honesty and that they will receive a bonus of $0.50 if their iscores are in the top 1/3rd of iscores for all responders in the experiment. The participants are not exposed to iscores while reporting their coin flips.

We then explain that the responder will be flipping a coin five times and reporting either heads or tails with each toss. Responders received a bonus of $0.01 for each heads that they report. After reporting the outcomes for each of the five coin flips, responders provide a prediction of the proportion of all coin flips by all participants in the experiment which were reported to be heads. This experiment assesses the improvement in honesty from BTS when the option to be honest (i.e. reporting tails) or dishonest (i.e. reporting heads) is relatively explicit.

#### Dice experiment

Responders in the Dice experiment are randomly assigned to control treatment (*N* = 1050), BTS intimidation (*N* = 1010), or transparent BTS (*N* = 947) when the survey webpage loads. Each responder receives a base pay of $0.20 for completing the survey. Responders in either BTS treatments were informed that an algorithm was in use to assess their honesty and that we will reward a bonus of $0.50 to responders who’s iscores were in the top 1/3rd of their treatment. Responders in the BTS intimidation group were not exposed to iscores, while responders in the transparent BTS treatment saw dynamically calculated iscores next to each option.

Responders then perform five dice rolls using six-sided dice while receiving a reward according to the dice roll outcomes they report. Rewards are calculated according to $0.01 × (sum of dice). This incentivizes responders to over report high dice outcomes. Finally, for each possible dice outcome (i.e. 1, 2, …, 6), each responder predicts the proportion of reported dice rolls with that outcome by all participants in the experiment. This experiment provides a range of options while still providing a clear incentive for dishonesty and a clear ground-truth distribution.

#### Pricing experiment

We use MTurk to assess the market value of the completion of a particular task, while incentivizing truthful and thoughtful responses. We created a twenty question multiple-choice questionnaire where participants are given a U.S. state and asked to select the state capital from a dropdown list of five American cities. Our interest is to assess which of the rewards from {$0.10, $0.20,…, $0.90, $1} is the appropriate reward for completing our questionnaire; this is analogous to assessing the market value of completing this questionnaire on the MTurk market place.

We reward responders a base pay of $0.20 for completing our survey. Responders are randomly assigned to the control treatment (*N* = 648), BTS intimidation treatment (*N* = 613), or transparent BTS treatment (*N* = 643). Responders in the BTS treatments are informed that the honesty of their responses will be measured using an algorithm and that we will reward a bonus of $1.50 to participants with iscores in the top 1/3rd of scores in their treatment at the completion of the experiment. Responders are then exposed to an example question from the questionnaire and asked to select the reward they would like to receive for completing the questionnaire of 20 such questions. If the responder is in the transparent BTS treatment, then the dynamically calculated iscores associated with each reward option are displayed next to the option. Next, responders predict the proportion of other participants who will select each of the reward options. Finally, participants are required to complete the questionnaire and are paid the reward amount which they selected. Responders in the control treatment maximize their personal gain by selecting the highest reward (i.e. $1) for completing the questionnaire.

## Results

**Fig 1:**
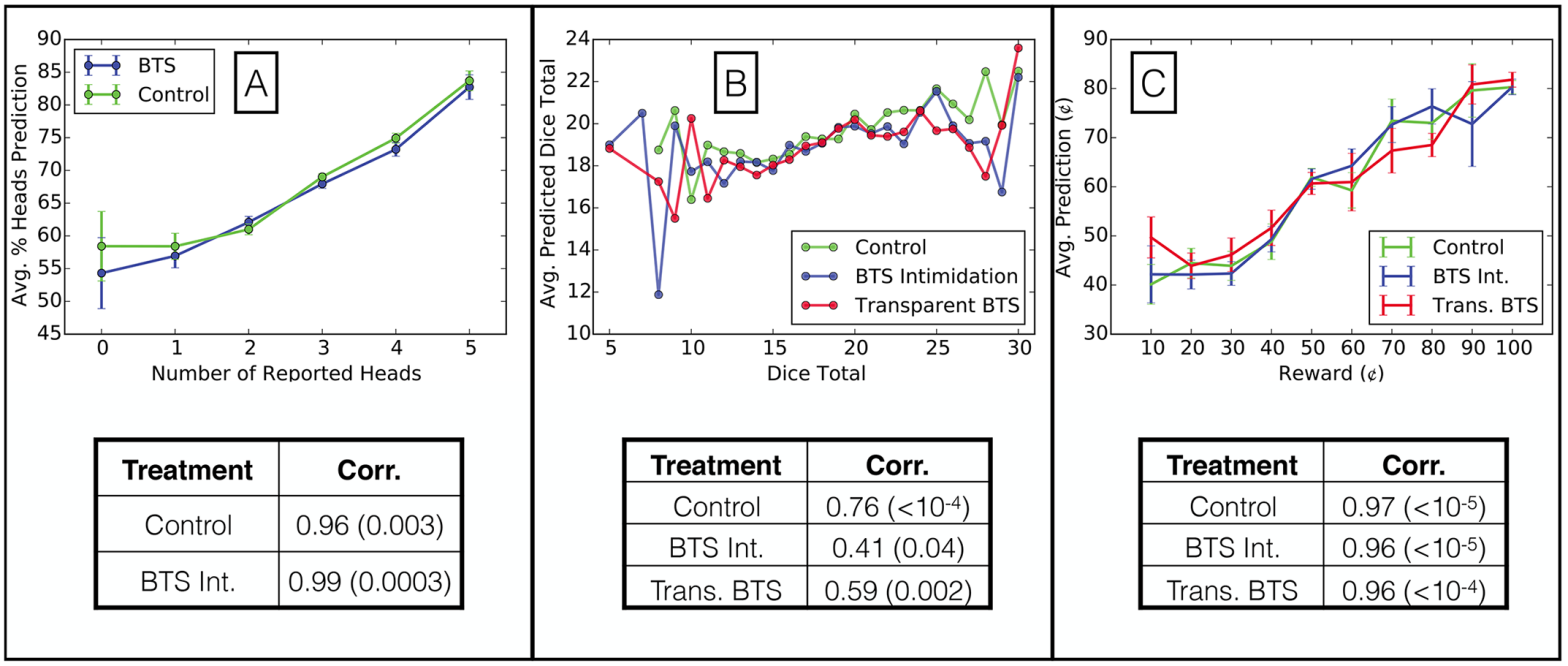
The Bayesian assumption of BTS holds in all treatments for each experiment. Along with each plot, we provide the Pearson correlation coefficient, and associated p-values in parentheses, between the endorsed responses and the predictions made by responders. **(A)** In the Coin Flip experiment, the average predicted percentage of heads increases with the number of heads reported by the responders. **(B)** In the Dice experiment, the average predicted dice total increases with the sum of the reported dice rolls from the responders. **(C)** In the Pricing experiment, the average predicted reward selected by responders for completing the questionnaire increases with the selected rewards of the responder.

The base assumption of BTS asserts that participants will disproportionately predict endorsements of their own beliefs, and this assumption holds for participants in all experimental treatments (see Fig 1). We have grouped responders according to their reported coin flips (Fig 1A), sum of reported dice outcomes (Fig 1B), and selected reward for task completion (Fig 1C), and, in each case, we find that predicted reward increases significantly with the responders’ selected reward. The more detailed distributions of predictions are available in S1 File. Since the base assumption is satisfied, we continue our investigation into the effects of BTS on honesty.

**Fig 2:**
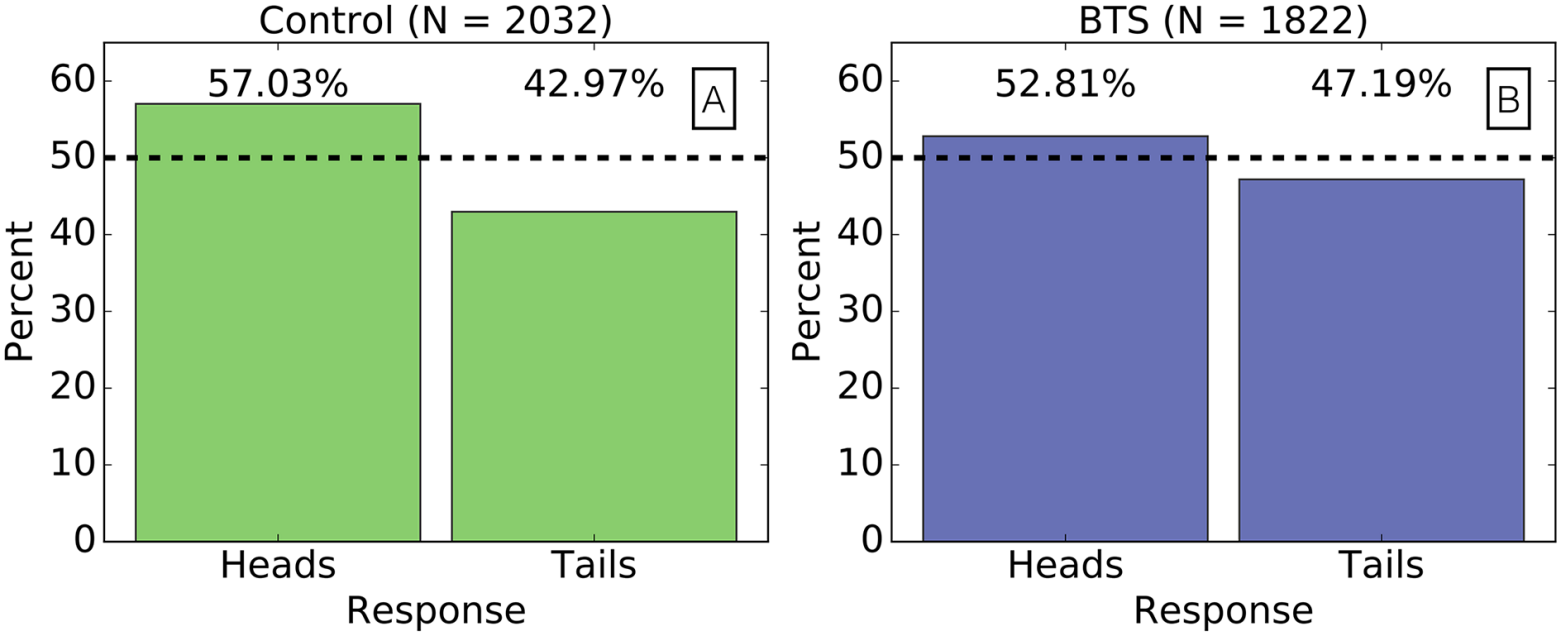
BTS improves honesty in the Coin Flip experiment. **(A)** 57% of reported coin flips were heads (43% tails) in the control treatment. **(B)** 53% of reported coin flips were heads (47% tails) in the BTS intimidation treatment. We expect 50% of coin flips to be heads if coin flips were reported honesty (represented by the black dashed line in both plots).

The Coin Flip experiment provided participants with only two choices with each coin toss; the choice to report a coin flip of tails received no additional payment and can therefore be considered as an indication of honesty by participants. The proportion of reported tails increased from 43% in the control treatment to 47% in the BTS treatment, while we expect 50% of reported coin flips to be tails if the coin flips were reported honestly (see Fig 2). This improvement in honesty is statistically significant according to the binomial statistical test (*p*_*val*_ < 10^−17^). This experimental design caused a relatively explicit decision about the honesty of responses, and so it remains to be seen how BTS performs when responders are given a broader range of options.

**Fig 3:**
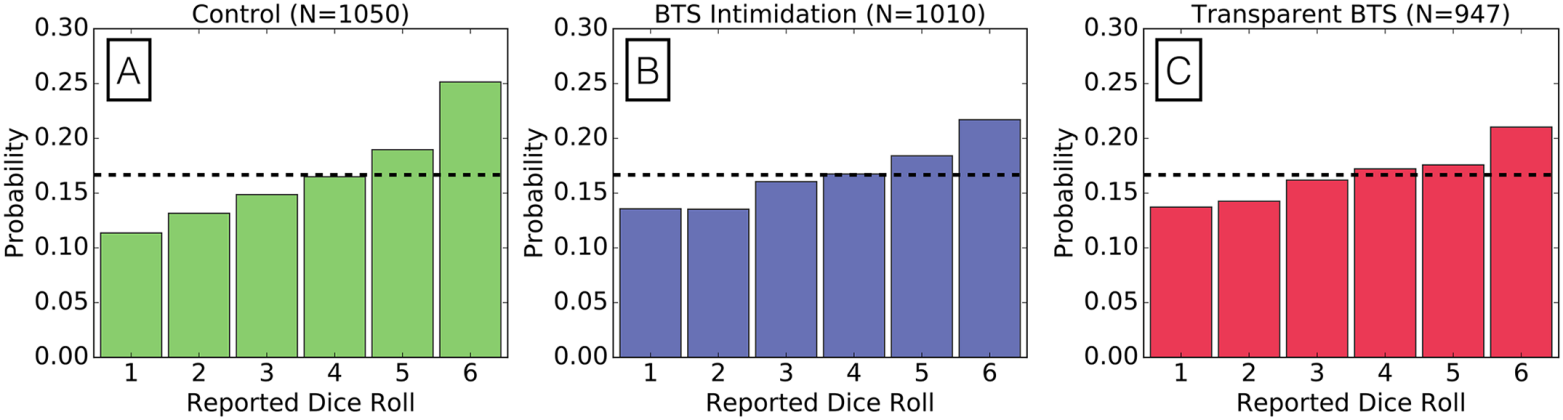
BTS treatments improve honesty in the Dice experiment. Probability mass function (PMF) of reported dice outcomes in **(A)** the control treatment, **(B)** BTS intimidation treatment, and **(C)** transparent BTS treatment. Honest reporting would produce a uniform distribution with values 1/6 in expectation (represented in each panel by the black dashed line).

The Dice experiment exposes participants to a broader range of options in terms of honesty. For example, a participant reporting that each of their dice rolls resulted in sixes is perhaps being dishonest since this outcome is statistically rare (*P*(all 6’s) = 1/6^5^) and profit maximizing, while a reported distribution of dice rolls including fours, fives, and sixes yields relatively high personal profit and is more likely to occur by chance (e.g. *P*(two 4’s, two 5’s, one 6) = 30/6^5^). Essentially, responders have a better ability to balance their profit-maximization with the believability of their response in this experiment by comparison to the Coin Flip experiment. Fig 3 demonstrates the probability mass functions of reported dice outcomes by treatment group. Both BTS treatments produced statistically significant improvements in honesty in comparison to the control treatment according to the Pearson’s *χ*^2^ goodness-of-fit test (*p*_*val*_ < 0.001, see Table 1). Transparent BTS, where the dynamically calculated iscores are present, did not exhibit a significant improvement over BTS intimidation, but produced the most “honest” distribution of dice outcomes.

**Table 1:** The Pearson’s *χ*^2^ goodness-of-fit statistic for pairwise distribution comparisons in the Dice experiment. Pairs of distributions are significantly different except for the distributions resulting from the two BTS treatments. Significance p-values are provided according to * = 0.1, ** = 0.01, and *** = 0.001.

	BTS Int.	Trans. BTS	Honest
Control	54.63***	82.46***	387.22***
BTS Int.		5.85	148.25***
Trans. BTS			101.02***

**Fig 4:**
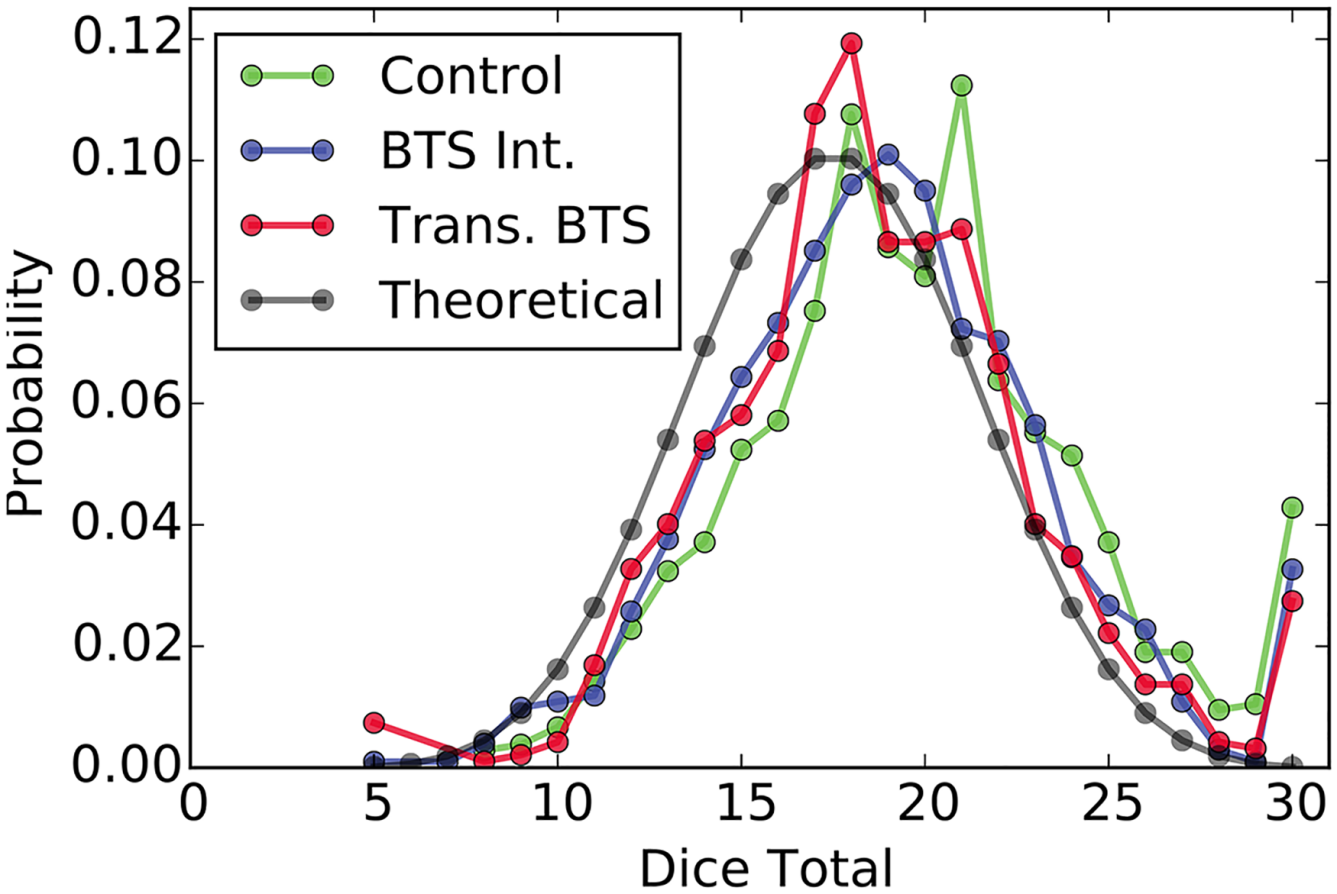
The distribution of the dice sums by treatment group. The expected distribution of dice totals if dice outcomes were reported honestly is presented in black.

Along with the distribution of dice outcomes, we assess the distribution of the sum of dice outcomes reported by responders in each treatment group in Fig 4. The distribution of dice totals in expectation under honest reporting is calculated according to

$$P\left(T\right)=\frac{1}{6^{5}}\overset{\left\lfloor \left(T-5\right)/6\right\rfloor }{\underset{k=0}{\sum }}{\left(-1\right)}^{k}\left(\begin{array}{l} 5\\ k \end{array}\right)\left(\begin{array}{l} T-6k-1\\ 4 \end{array}\right),$$

where *T* = 5, 6, …, 30 denotes the dice total. Each experimental distribution of dice totals is significantly distinguishable from the honest distribution according to the Pearson goodness-of-fit test (see Table 2). The distributions resulting from the BTS treatments each indicate that the collection of dice outcomes reported by individual responders produced increasingly honest looking dice totals on aggregate despite the apparent dishonesty when viewing the aggregate distributions of individual dice outcomes (see Fig 3). Still, each distribution of dice totals is shifted to the right of the honest distribution and therefore highlights the bias towards profit-maximization as individual responders balance there personal profit with the believability of their reported dice outcomes. The sharp increases at the right end of each empirical distribution indicates that there were responders in each treatment who reported rolling all sixes despite the low probability of that particular outcome by chance. However, the number of participants selecting to report this outcome decreased in the BTS treatments in comparison to the control treatment.

**Table 2:** BTS produces statistically distinguishable distributions of dice totals. The table displays the Pearson’s *χ*^2^ goodness-of-fit statistic for pairwise distribution comparisons. Each pair of distributions was significantly different from each other. Significance p-values are provided according to * = 0.1, ** = 0.01, and *** = 0.001.

	BTS Int.	Trans. BTS	Honest
Control	185.15***	108.46***	15332.94***
BTS Int.		98.56***	8416.23***
Trans. BTS			5979.77***

The Dice experiment demonstrates that BTS remains effective at combating dishonesty when responders have a diversified ability to balance the believability and the profit-maximization of their responses. Real-world surveys, such as product satisfaction surveys, rarely have known underlying “honest” distributions. After all, if the true distribution were known, then why run the survey? This observation makes it difficult to validate the honesty or usefulness of real-world survey responses in general. However, we have demonstrated that BTS improves honesty in simple experiments, and we endeavor to discover the effects of BTS in a more realistic application.

The Pricing experiment described a task to responders and asked them to select a reward for their completion of that task. This experiment is designed to assess the MTurk market value for the completion of the task and is analogous to using online survey for assessing the market value of a product according to consumers. Marketing departments are constantly performing these surveys, but obtaining thoughtful or truthful results can be difficult as it is not obvious how to incentivize or measure the effort given to responses. In our experiment, responders are motivated to select higher rewards to maximize their personal profit for completing the task, while actual product pricing surveys incentivize responders to select lower values so as to lower the retail price of the product. Up to symmetry, this subtle difference is immaterial to the performance of BTS.

**Fig 5:**
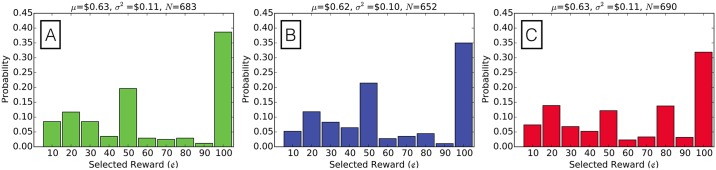
BTS treatments alter reward selection in comparison to the control treatment. We present the probability mass function of selected rewards for task completion in the **(A)** control treatment, **(B)** BTS intimidation treatment, and the **(C)** transparent BTS treatment.

BTS has a significant effect in the Pricing experiment without changing task performance (see Section 4 in S1 File). Fig 5 displays the proportion of responders selecting each reward for the completion of the task according to treatment. Each pair of distributions is significantly different according to the Pearson’s *χ*^2^ goodness-of-fit test (see Table 3). In particular, the transparent BTS distribution appears to be most dissimilar from the other distributions with an additional peak representing an increase in responders willing to complete the task for a reward of $0.80 along with a diminished peak at the $0.50 reward. Additionally, the proportion of responders who (perhaps greedily) selected to complete the task for the maximum reward of $1 diminished in the BTS treatments in comparison to the control treatment. As discussed, it is difficult to validate these changes by comparison to some “honest” ground-truth distribution. Nevertheless, BTS verifiably improved responses in the Coin Flip and Dice experiments, and, therefore, suggests that the changes we observe in the Pricing experiment are improvements in information quality as well.

**Table 3:** The pairwise Pearson’s *χ*^2^ goodness-of-fit statistic comparing selected rewards in the Pricing experiment. Each pair of distributions is significantly different. Significance p-values are provided according to * = 0.1, ** = 0.01, and *** = 0.001.

	BTS Int.	Trans. BTS
Control	31.37***	110.41***
BTS Int.		94.23***

## Discussion

Our experiments demonstrate that Bayesian truth serum (BTS) indeed improves responses to large-scale online survey. We performed our experiments with a realistic number of participants in comparison to real-world marketing applications. The Coin Flip experiment and the Dice experiment demonstrate the effectiveness of BTS in combating dishonesty in simple scenarios where the “honest” distribution is known in expectation. Since certain outcomes were explicitly incentivized, it was perhaps clear to participants that their honesty was the focus of our investigation. Despite this concern, we find evidence for dishonest behavior in each experiment and find that BTS diminishes this dishonest behavior.

One might wonder if the improvements from BTS are instead due to increased payment in expectation for participants in the BTS treatments compared to participants in the control treatment. Existing work has investigating the effects of participation rewards on survey quality and found that increased financial incentives increases the quantity of work performed by participants but does not increase the quality of the work [40]. In agreement with this finding, we ran alternative control treatments for all three experiments and found that honesty was not increased as a result of increased participant pay in the control treatment to match the pay of participants in BTS treatments in expectation (see Section 5 in S1 File). Therefore, we may conclude that changes across treatments are not a result of slight differences in participation rewards.

In experiments where the ground-truth was known, BTS led to improvements in honesty from responders. BTS also produced statistically different results in a real-world application assessing the market value of a task. However, as with most real-world surveys, it is difficult to identify a ground-truth with which to compare distributions of responses. Nevertheless, BTS decreased the proportion of responders selecting the largest reward for task completion, and the transparent BTS treatment produced considerably different results from both the control treatment and the BTS intimidation treatment. Combining these observations with our results from the Coin Flip and Dice experiments leads us to conclude that BTS indeed improves the honesty and thoughtfulness of responses in online surveys with one-sided incentives.

BTS works because of the Bayesian assertion that people conceptualize models of the world which are influenced heavily by their own experience. In particular, we find that the reward selected by responders is very indicative of their predictions about the actions of other participants across all treatments and all experiments. This observation about human nature appears to be fairly ubiquitous, and therefore suggests that BTS is applicable to a wide range of survey applications and a wide range of responder populations.

Competing methods to combat dishonesty and apathy in online survey include agreeing to an honesty pledge or invoking religious faith. Effectively, survey requesters are attempting to instill guilt into would-be dishonest responders. In our experiments, BTS intimidation has much the same effect as no quantifiable evidence of the BTS algorithm is presented to responders while they select their responses. Therefore, it may be that improvements in honesty from the BTS intimidation treatment are due to nothing more than the threat of an honesty measuring algorithm, and similar improvements could perhaps be obtained through the threat of an angry god or loss of personal integrity. The transparent BTS treatment in our Dice experiment and Pricing experiment exposed responders to dynamically calculated iscores as the responders made their selections. Responders were actually subject to quantified influence from the BTS algorithm, and our results suggest that this additional influence improves survey results further. In particular, the transparent BTS treatment in the Pricing experiment reveals a previously unseen class of responders willing to accept a reward of $0.80 for the completion of the questionnaire and reduces the proportion of responders selecting the largest reward of $1 for task completion. The ability to quantify information quality through the iscore calculation allows BTS to be more effective than traditional survey techniques.

In all three experiments, BTS was used to successfully combat a one-sided monetary bias (i.e. all participants increase personal profit by selecting the same responses). BTS has been suggested as a tool for improving all subjective survey response in the literature, but it remains to investigate how BTS performs when contrary incentive structures exist within the responder population. For example, consider surveying democrats and republicans about a politically polarized issue; innate political biases may influence the performance of BTS. This observation motivates us to seek out scenarios where BTS may under perform and to identify appropriate countermeasures.

Finally, validation is the greatest challenge facing investigations into survey methods. Our Coin Flip and Dice experiments innately contain ground-truth distributions representing honesty in these contexts, but these ground-truth distributions are typically absent in real-world survey applications. Advancements in survey methods and their validation rely on the willingness of researchers and companies employing online survey to share data and insight. This cooperation will address an issue we came across in our study and lead to future improvements in online survey technique.

## Supporting information

S1 File Supplementary information. (PDF)
