# Supplementary material for: Validating Bayesian truth serum in large-scale online human experiments
Source: PLoS One. 2017 May 11;12(5):e0177385. doi: 10.1371/journal.pone.0177385 (PMC5426759; doi:10.1371/journal.pone.0177385)
Supplement: S1 File — (PDF) [file pone.0177385.s001.pdf]

Validating Bayesian Truth Serum in Large-scale Online Human Experiments

Morgan R. Frank[1], Manuel Cebrian[1,2], Galen Pickard[3], Iyad Rahwan[1,*]

[1]Media Laboratory, Massachusetts Institute of Technology, Cambridge, MA, USA
[2]Data61 Unit, Commonwealth Scientific and Industrial Research Organization, Melbourne, Victoria, Australia
[3]Google Inc., Mountain View, CA, USA
[*]corresponding author: irahwan@mit.edu

Contents

1 Predicted Distributions: Coin Flip Experiment 2

2 Predicted Distributions: Dice Experiment 5

3 Predicted Distributions: Marketing Experiment 7

4 Questionnaire Performance by Selected Reward in the Marketing Experiment 7

5 Increasing Payment Expectation Does Not Increase Honesty 11

6 Screen Shots of Experiments 12
6.1 Coin Flip Experiment 12
6.2 Dice Experiment 15
6.3 Pricing Experiment 17

1 Predicted Distributions: Coin Flip Experiment

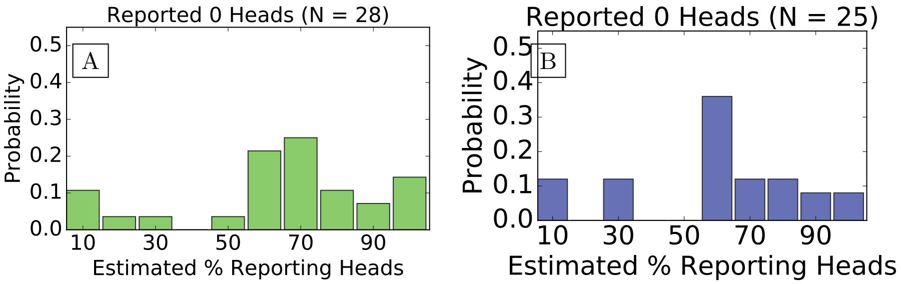

Figure 1: Distribution of predicted rates of coin flips reported as heads from responders who themselves reported 0 heads. **(A)** Distribution of predictions for responders in the control treatment. **(B)** Distribution of predictions for responders in the BTS intimidation treatment.

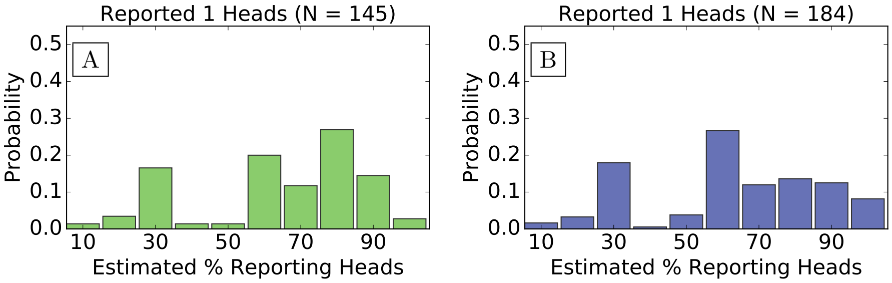

Figure 2: Distribution of predicted rates of coin flips reported as heads from responders who themselves reported 1 heads. **(A)** Distribution of predictions for responders in the control treatment. **(B)** Distribution of predictions for responders in the BTS intimidation treatment.

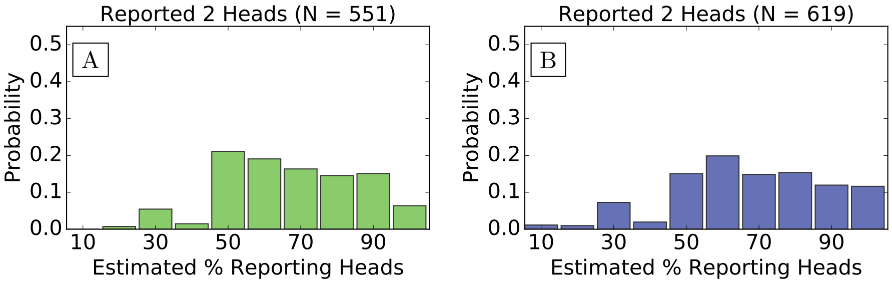

Figure 3: Distribution of predicted rates of coin flips reported as heads from responders who themselves reported 2 heads. **(A)** Distribution of predictions for responders in the control treatment. **(B)** Distribution of predictions for responders in the BTS intimidation treatment.

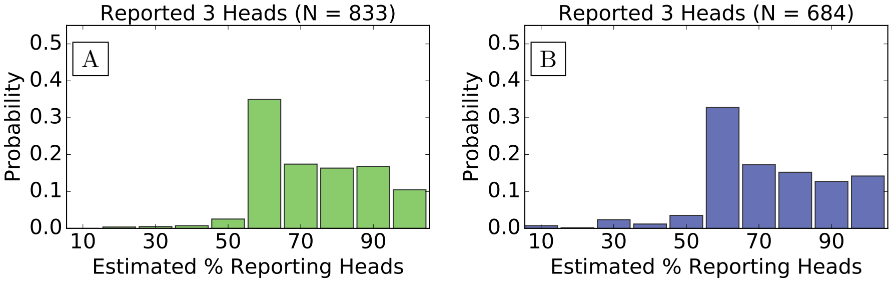

Figure 4: Distribution of predicted rates of coin flips reported as heads from responders who themselves reported 3 heads. **(A)** Distribution of predictions for responders in the control treatment. **(B)** Distribution of predictions for responders in the BTS intimidation treatment.

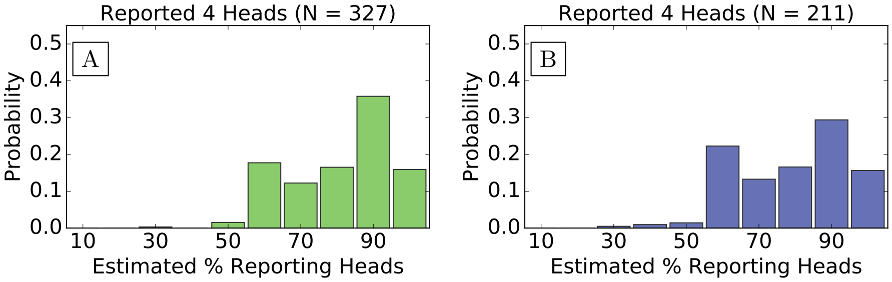

Figure 5: Distribution of predicted rates of coin flips reported as heads from responders who themselves reported 4 heads. **(A)** Distribution of predictions for responders in the control treatment. **(B)** Distribution of predictions for responders in the BTS intimidation treatment.

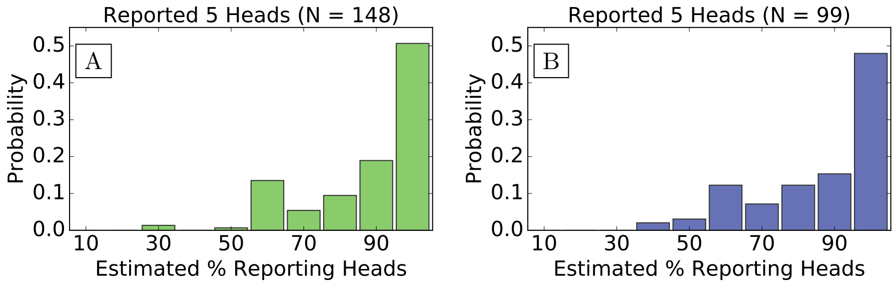

Figure 6: Distribution of predicted rates of coin flips reported as heads from responders who themselves reported 5 heads. **(A)** Distribution of predictions for responders in the control treatment. **(B)** Distribution of predictions for responders in the BTS intimidation treatment.

2 Predicted Distributions: Dice Experiment

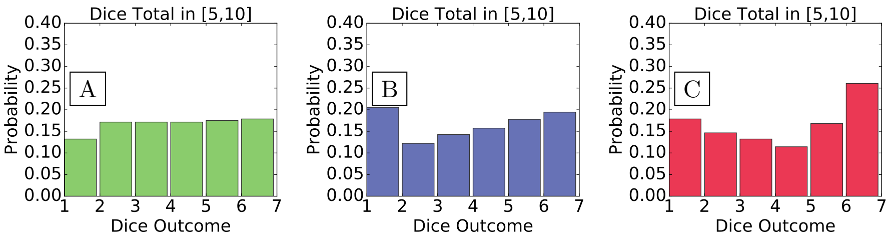

Figure 7: Predicted distributions of dice outcomes by responder dice total and treatment. **(A)** Control Treatment. **(B)** BTS intimidation Treatment. **(C)** Transparent BTS Treatment.

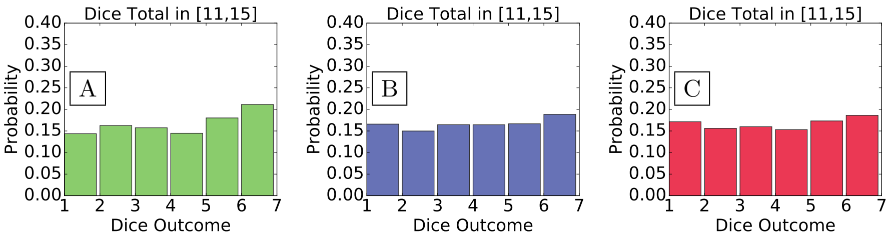

Figure 8: Predicted distributions of dice outcomes by responder dice total and treatment. **(A)** Control Treatment. **(B)** BTS intimidation Treatment. **(C)** Transparent BTS Treatment.

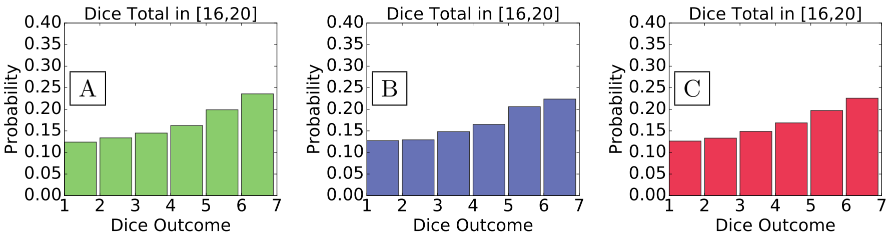

Figure 9: Predicted distributions of dice outcomes by responder dice total and treatment. **(A)** Control Treatment. **(B)** BTS intimidation Treatment. **(C)** Transparent BTS Treatment.

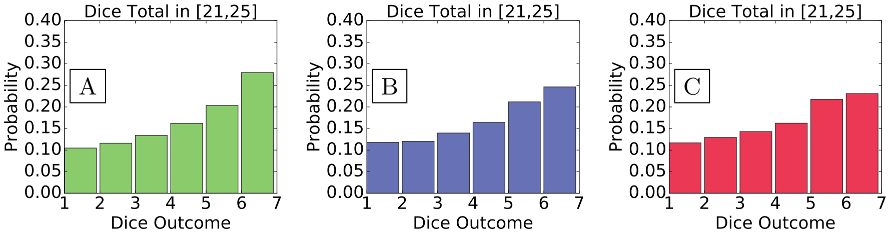

Figure 10: Predicted distributions of dice outcomes by responder dice total and treatment. **(A)** Control Treatment. **(B)** BTS intimidation Treatment. **(C)** Transparent BTS Treatment.

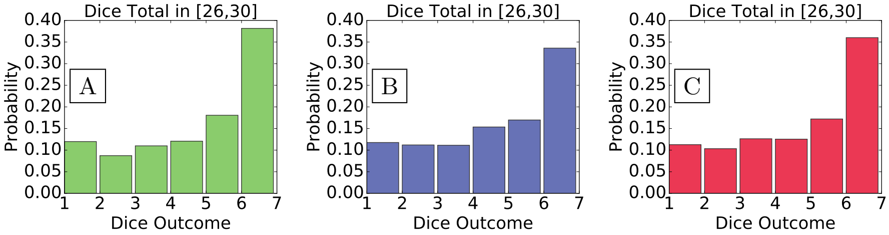

Figure 11: Predicted distributions of dice outcomes by responder dice total and treatment. **(A)** Control Treatment. **(B)** BTS intimidation Treatment. **(C)** Transparent BTS Treatment.

3 Predicted Distributions: Marketing Experiment

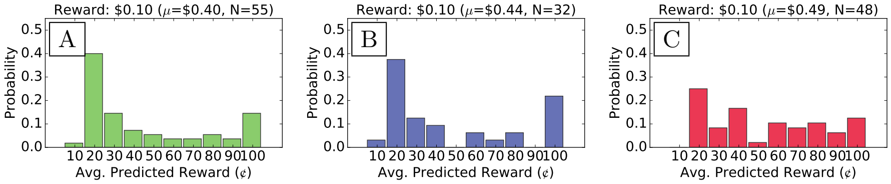

Figure 12: Predicted distributions of selected task rewards from responders selecting a reward of 10¢. **(A)** Control Treatment. **(B)** BTS intimidation Treatment. **(C)** Transparent BTS Treatment.

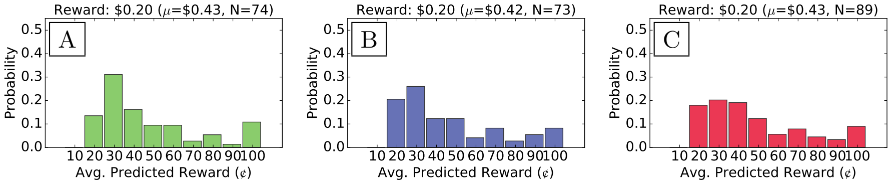

Figure 13: Predicted distributions of selected task rewards from responders selecting a reward of 20¢. **(A)** Control Treatment. **(B)** BTS intimidation Treatment. **(C)** Transparent BTS Treatment.

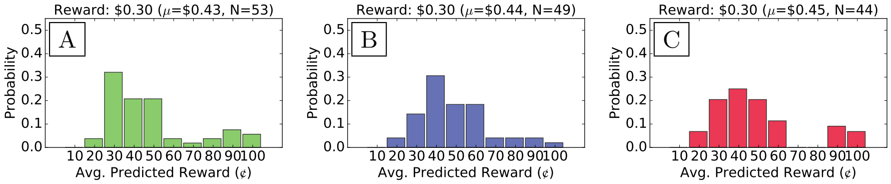

Figure 14: Predicted distributions of selected task rewards from responders selecting a reward of 30¢. **(A)** Control Treatment. **(B)** BTS intimidation Treatment. **(C)** Transparent BTS Treatment.

4 Questionnaire Performance by Selected Reward in the Marketing Experiment

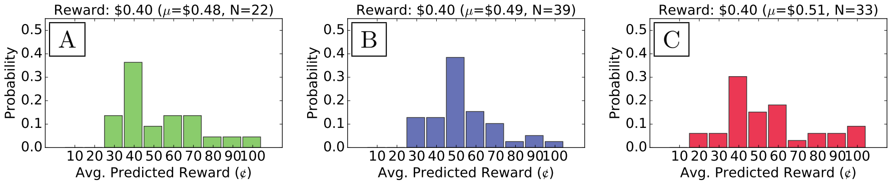

Figure 15: Predicted distributions of selected task rewards from responders selecting a reward of 40¢. **(A)** Control Treatment. **(B)** BTS intimidation Treatment. **(C)** Transparent BTS Treatment.

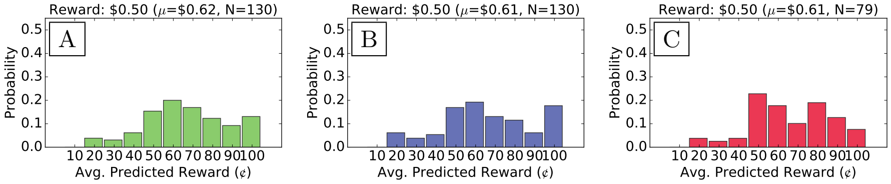

Figure 16: Predicted distributions of selected task rewards from responders selecting a reward of 50¢. **(A)** Control Treatment. **(B)** BTS intimidation Treatment. **(C)** Transparent BTS Treatment.

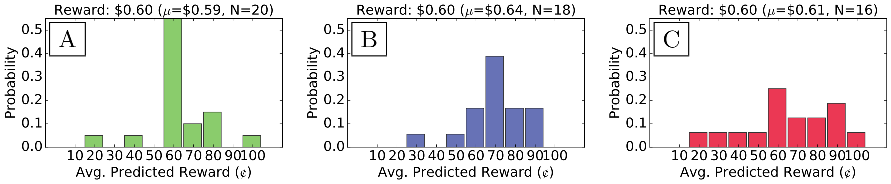

Figure 17: Predicted distributions of selected task rewards from responders selecting a reward of 60¢. **(A)** Control Treatment. **(B)** BTS intimidation Treatment. **(C)** Transparent BTS Treatment.

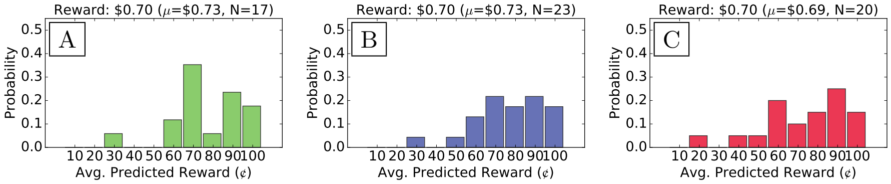

Figure 18: Predicted distributions of selected task rewards from responders selecting a reward of 70¢. **(A)** Control Treatment. **(B)** BTS intimidation Treatment. **(C)** Transparent BTS Treatment.

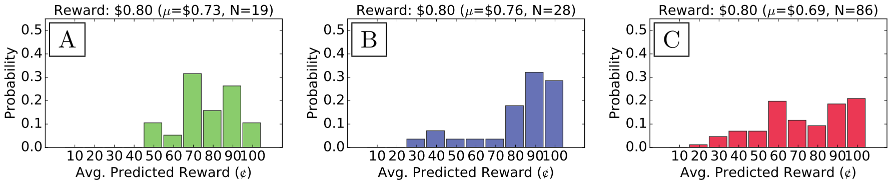

Figure 19: Predicted distributions of selected task rewards from responders selecting a reward of 80¢. **(A)** Control Treatment. **(B)** BTS intimidation Treatment. **(C)** Transparent BTS Treatment.

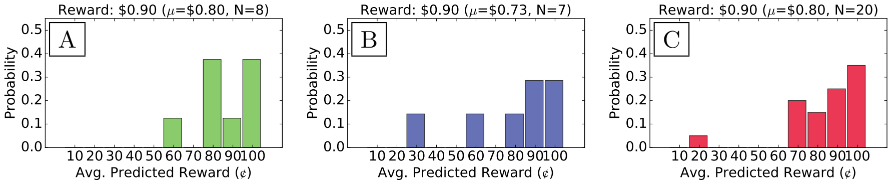

Figure 20: Predicted distributions of selected task rewards from responders selecting a reward of 90¢. **(A)** Control Treatment. **(B)** BTS intimidation Treatment. **(C)** Transparent BTS Treatment.

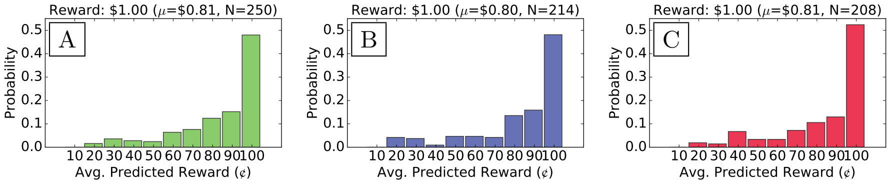

Figure 21: Predicted distributions of selected task rewards from responders selecting a reward of 100¢. **(A)** Control Treatment. **(B)** BTS intimidation Treatment. **(C)** Transparent BTS Treatment.

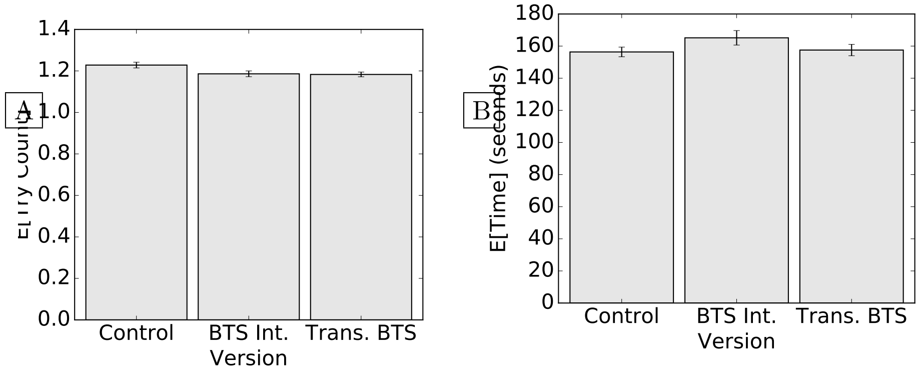

Figure 22: Questionnaire performance is not appreciably altered by BTS treatments in the Marketing experiment. **(A)** The average number of attempts to correctly answer individual questions in the questionnaire. **(B)** The average completion time for the questionnaire.

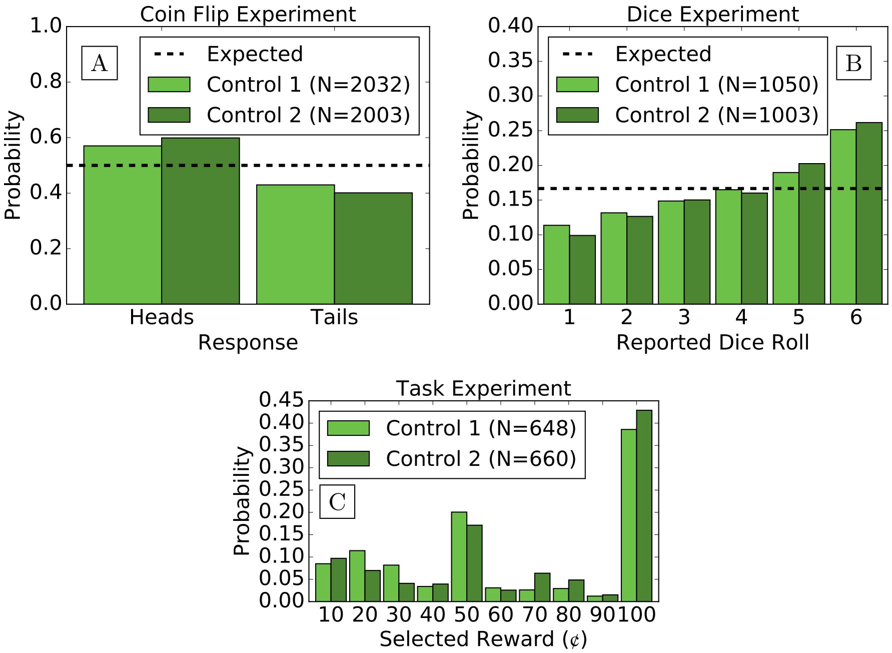

Figure 23: Examining the effects of increased payment on honesty in control treatment. For the **(A)** coin flip experiment, **(B)** the dice experiment, and **(C)** the task experiment, the response from control treatments as described in the main text are denoted "Control 1", while "Control 2" represents the response from an alternative payment scheme where participants make the same money for participation as participants in BTS treatments in expectation.

5 Increasing Payment Expectation Does Not Increase Honesty

Existing work has investigating the effects of participation rewards on survey quality and found that increased financial incentives increases the quantity of work performed by participants but does not increase the quality of the work [1]. Yet, we wonder if the difference between control and BTS treatments that we observe are simply a result of increased payment in expectation in the BTS treatments that results from the BTS rewards (independent of response and prediction rewards). We test this by running another control treatment (i.e. no BTS reward or instruction) where the base pay is equal to the reward in expectation for participants in BTS treatments (i.e. base pay from original control treatment plus 1/3rd the BTS reward in the corresponding BTS treatments).

Figure 23 presents the resulting response distributions from the control treatment with increased base pay (denoted "Control 2") for comparison to the control treatment described in the main text (denoted "Control 1"). In each case,

the response distribution for Control 2 is statistically significantly different from the Control 1 distribution (i.e. $p_{val} < 0.001$) according to the binomial statistic for the coin experiment, and according to the Pearson goodness-of-fit statistic for the dice experiment and the pricing experiment. Interestingly, participants in the Control 2 treatment of each experiment were more likely to select the response yielding maximum reward compared to participants in the Control 1 treatment. We conclude that the change in response and improvements in honesty which result from the BTS treatments in each experiment are not the result of increased pay in expectation (in good agreement with [1]).

6 Screen Shots of Experiments

In this section, we provide descriptions of the participant experience along with screen shots from the online survey for the coin flip, dice, and pricing experiments.

6.1 Coin Flip Experiment

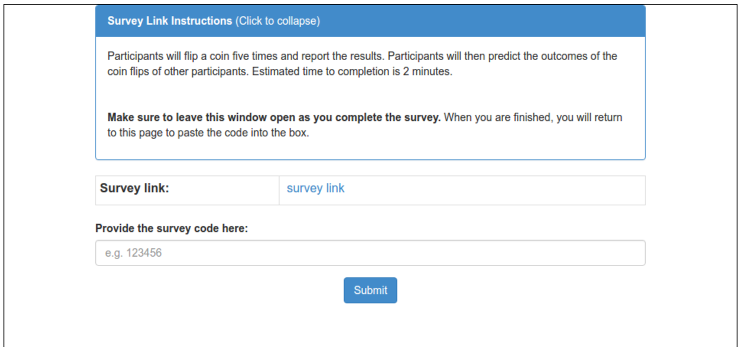

Figure 24: Amazon's Mechanical Turk Survey Link instructions for potential survey participants.

Participants for the Coin Flip experiment are recruited using Amazon's Mechanical Turk (MTurk). Figure 24 demonstrates the instructions provided to participants when selecting to participate. At the completion of the survey, we generate a unique survey key which participants submit to MTurk at this page to demonstrate their completion. This code also allows us to award bonus payments to participants from their selected responses and from BTS rewards.

Once participants agree to participate via MTurk, they follow a hyperlink to the survey webpage. Once on the survey webpage, participants are exposed to each of four panels of the survey one at a time. First, participants provide some basic demographic information including gender, state of residence, industry of employment, and age from drop down menus (see Fig. 25). A submit button appears once participants update each field.

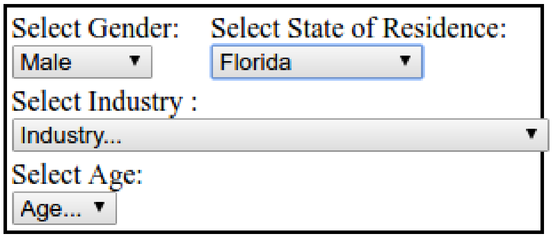

Figure 25: Each experiment begins with a four-question demographics survey.

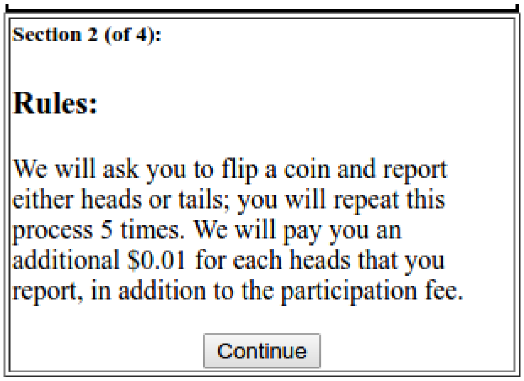

Figure 26: Coin flip experiment instructions for all treatments.

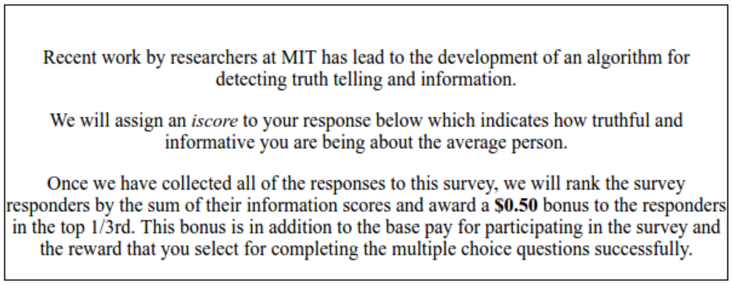

Figure 27: Coin flip experiment instructions regarding BTS rewards. These instructions were only provided to participants in the BTS treatment.

Figure 26 demonstrates the description of rewards for coin flip reporting for participants in either treatment. Participants select the Continue button to advance. Participants in the BTS treatment were then subject to additional instructions describing the BTS reward based on iscores (see Fig. 27); after ten seconds, a Continue button appears which allows participants to advance from here.

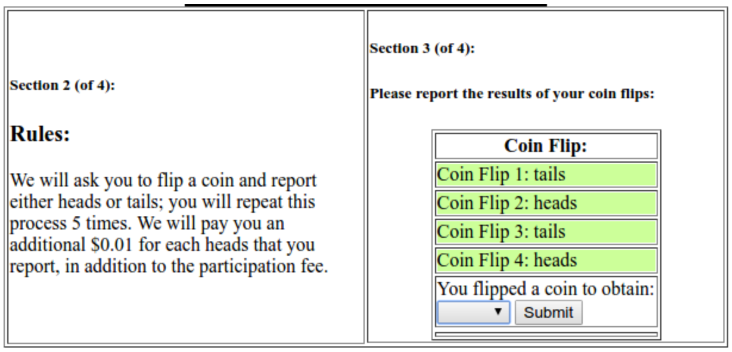

Figure 28: Participants report the results of five coin flips using drop down menus.

Depicted in Figure 28, participants report the results of their coin flips using drop down menus. Drop down options include “heads” or “tails” and the order in which these options are presented is randomized for each drop down menu. Participants commit to their drop down menu selection by pressing the Submit button, after which the space occupied by the drop down menu is replaced by the participants selection.

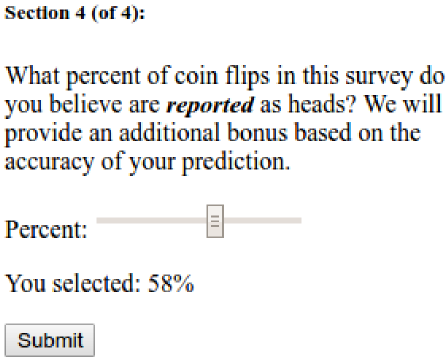

Figure 29: Participants predict the percent of coin flips reported as heads by all participants in the experiment using a slider.

Finally, participants predict the proportion of coin flips from all participants in the experiment that will be reported as heads (see Fig. 29). Participants are informed that they will receive an additional reward based on the accuracy of their prediction. Figure 30 demonstrates an example completed survey webpage. A unique survey key is generated for each participant who successfully completes the survey which the participant reports to MTurk.

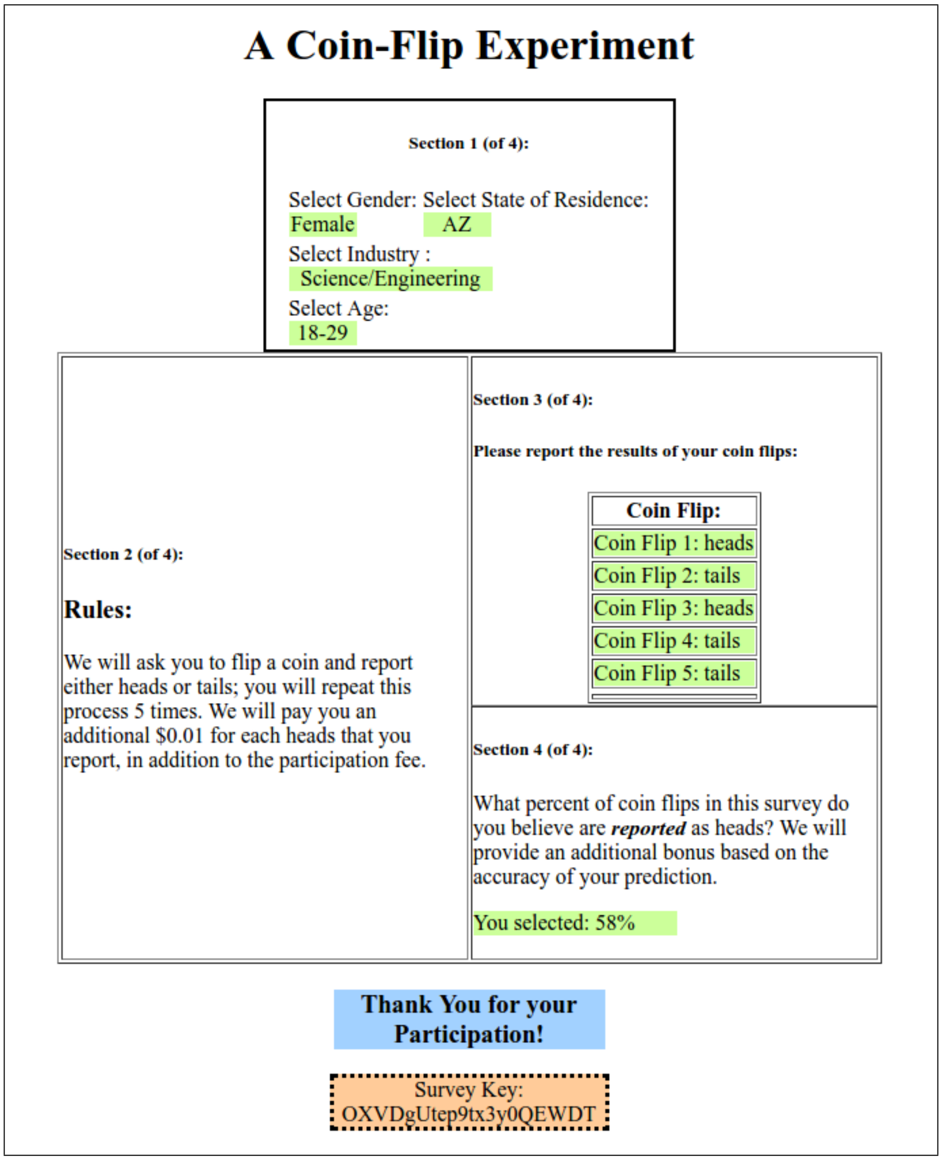

Figure 30: An example completed survey webpage for the Coin Flip experiment.

6.2 Dice Experiment

Participants for the Dice experiment are recruited using Amazon's Mechanical Turk (MTurk). Figure 31 demonstrates the instructions provided to participants when selecting to participate. At the completion of the survey, we generate a unique survey key which participants submit to MTurk at this page to demon-

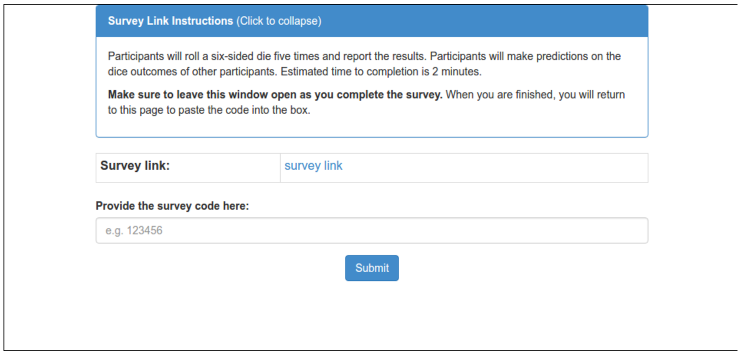

Figure 31: Amazon's Mechanical Turk Survey Link instructions for potential survey participants.

strate their completion. This code also allows us to award bonus payments to participants from their selected responses and from BTS rewards.

Once participants agree to participate via MTurk, they follow a hyperlink to the survey webpage. Once on the survey webpage, participants are exposed to each of four panels of the survey one at a time. First, participants provide some basic demographic information including gender, state of residence, industry of employment, and age from drop down menus (see Fig. 25). A submit button appears once participants update each field.

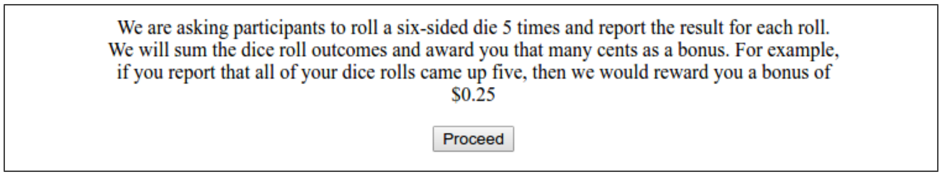

Figure 32: Dice experiment instructions for all treatments.

Figure 32 demonstrates the description of rewards for dice reporting for participants in any treatment. Participants select the Continue button to advance. Participants in the BTS treatment were then subject to additional instructions describing the BTS reward based on iscores (see Fig. 33); after ten seconds, a Continue button appears which allows participants to advance from here.

Depicted in Figure 34, participants report the results of their die rolls using drop down menus. Drop down options include 1,2,3,4,5, or 6 presented in increasing order. Participants commit to their drop down menu selections by pressing the Submit button, after which the spaces occupied by each drop down menu is replaced by the participants selection for that drop down menu.

Finally, participants predict the proportion of die rolls from all participants in the experiment that will be reported as 1,2,3,4,5, or 6 (see Fig. 35). Predictions submitted by each participant must sum to 100%. Participants are

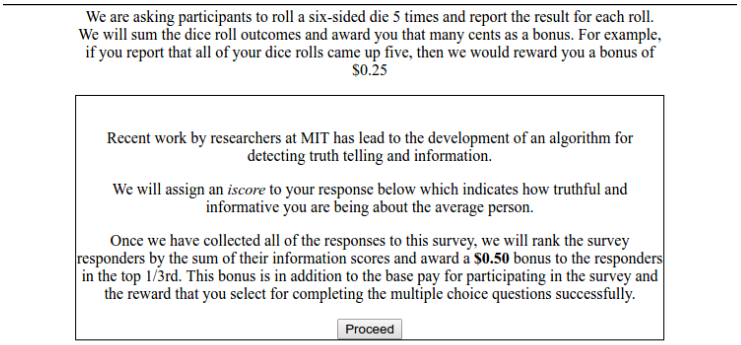
We are asking participants to roll a six-sided die 5 times and report the result for each roll. We will sum the dice roll outcomes and award you that many cents as a bonus. For example, if you report that all of your dice rolls came up five, then we would reward you a bonus of $0.25

Recent work by researchers at MIT has lead to the development of an algorithm for detecting truth telling and information.

We will assign an *iscore* to your response below which indicates how truthful and informative you are being about the average person.

Once we have collected all of the responses to this survey, we will rank the survey responders by the sum of their information scores and award a **$0.50** bonus to the responders in the top 1/3rd. This bonus is in addition to the base pay for participating in the survey and the reward that you select for completing the multiple choice questions successfully.

Proceed

Figure 33: Dice experiment instructions regarding BTS rewards. These instructions were only provided to participants in the BTS treatments.

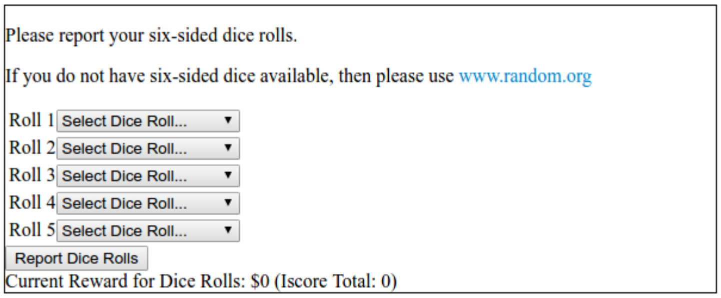
Please report your six-sided dice rolls.

If you do not have six-sided dice available, then please use www.random.org

Roll 1 Select Dice Roll...
Roll 2 Select Dice Roll...
Roll 3 Select Dice Roll...
Roll 4 Select Dice Roll...
Roll 5 Select Dice Roll...
Report Dice Rolls
Current Reward for Dice Rolls: $0 (Iscore Total: 0)

Figure 34: Participants report the results of five rolls of a six-sided die using drop down menus.

informed that they will receive an additional reward based on the accuracy of their prediction. Figure 36 demonstrates an example completed survey webpage. A unique survey key is generated for each participant who successfully completes the survey which the participant reports to MTurk.

6.3 Pricing Experiment

Participants for the Pricing experiment are recruited using Amazon's Mechanical Turk (MTurk). Figure 37 demonstrates the instructions provided to participants when selecting to participate. At the completion of the survey, we generate a unique survey key which participants submit to MTurk at this page to demonstrate their completion. This code also allows us to award bonus payments to participants from their selected responses and from BTS rewards.

Once participants agree to participate via MTurk, they follow a hyperlink to the survey webpage. Once on the survey webpage, participants are exposed to each of four panels of the survey one at a time. First, participants provide some basic demographic information including gender, state of residence, industry of

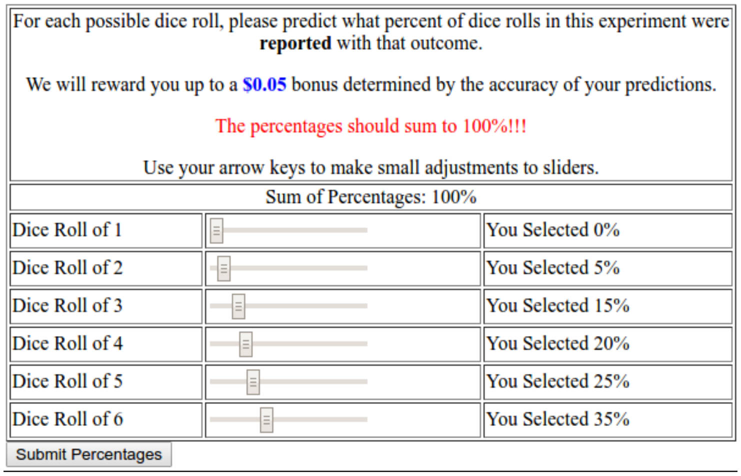

Figure 35: Participants predict the percent of die rolls reported as 1,2,3,4,5, or 6 by all participants in the experiment using sliders. Note that predictions must sum to 100% for the Submit button to appear.

employment, and age from drop down menus (see Fig. 25). A submit button appears once participants update each field.

Figure 38 demonstrates the instructions to participants. All participants are subject to a description of the questionnaire with an example question. Participants advance by selecting a Continue button. Participants in the BTS treatments were then subject to additional instructions describing the BTS reward based on iscores (see Fig. 33); after ten seconds, a Proceed button appears which allows participants to advance from here.

Depicted in Figure 39, participants select the reward they will receive for the completion of the questionnaire using a drop down menu. Participants commit to their drop down menu selection by pressing the Select button.

Next, participants predict the percent of participants selecting each reward for questionnaire completion (see Fig. 40). Predictions submitted by each participant must sum to 100%. Participants are informed that they will receive an additional reward based on the accuracy of their prediction.

Finally, participants complete the 20 mulitple-choice questionnaire (see Fig. 41 for an example question). Participants select options until the correct option is selected. Once participants correctly answer each question, we generate a unique survey code which the participants report to MTurk.

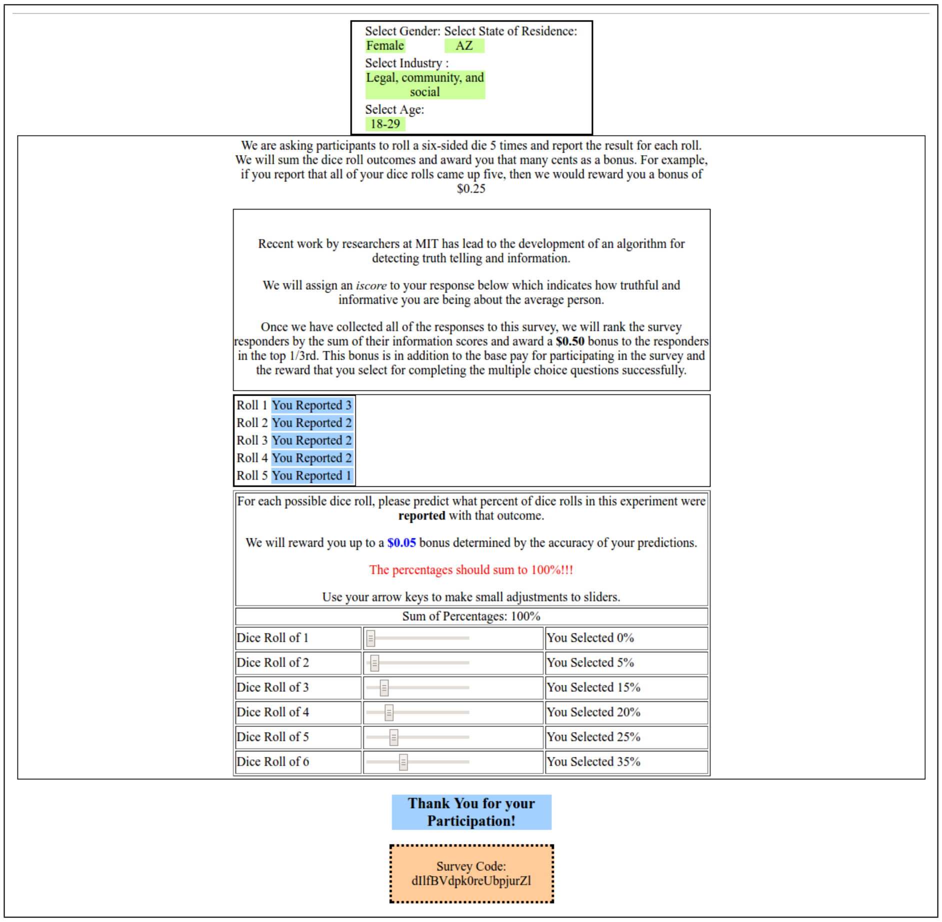

Select Gender: Select State of Residence:
Female AZ
Select Industry :
Legal, community, and social
Select Age:
18-29

We are asking participants to roll a six-sided die 5 times and report the result for each roll. We will sum the dice roll outcomes and award you that many cents as a bonus. For example, if you report that all of your dice rolls came up five, then we would reward you a bonus of $0.25

Recent work by researchers at MIT has lead to the development of an algorithm for detecting truth telling and information.

We will assign an *iscore* to your response below which indicates how truthful and informative you are being about the average person.

Once we have collected all of the responses to this survey, we will rank the survey responders by the sum of their information scores and award a **$0.50** bonus to the responders in the top 1/3rd. This bonus is in addition to the base pay for participating in the survey and the reward that you select for completing the multiple choice questions successfully.

Roll 1	You Reported 3
Roll 2	You Reported 2
Roll 3	You Reported 2
Roll 4	You Reported 2
Roll 5	You Reported 1

For each possible dice roll, please predict what percent of dice rolls in this experiment were **reported** with that outcome.

We will reward you up to a **$0.05** bonus determined by the accuracy of your predictions.

The percentages should sum to 100%!!!

Use your arrow keys to make small adjustments to sliders.

Sum of Percentages: 100%

Dice Roll of 1		You Selected 0%
Dice Roll of 2		You Selected 5%
Dice Roll of 3		You Selected 15%
Dice Roll of 4		You Selected 20%
Dice Roll of 5		You Selected 25%
Dice Roll of 6		You Selected 35%

Thank You for your Participation!

Survey Code: dIlfBVdpk0reUbpjurZl

Figure 36: An example completed survey webpage for the Dice experiment.

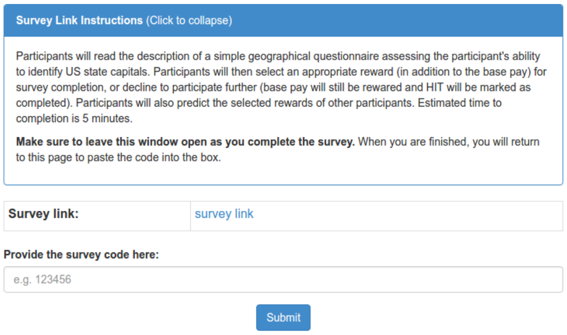

Survey Link Instructions (Click to collapse)

Participants will read the description of a simple geographical questionnaire assessing the participant's ability to identify US state capitals. Participants will then select an appropriate reward (in addition to the base pay) for survey completion, or decline to participate further (base pay will still be rewarded and HIT will be marked as completed). Participants will also predict the selected rewards of other participants. Estimated time to completion is 5 minutes.

Make sure to leave this window open as you complete the survey. When you are finished, you will return to this page to paste the code into the box.

Survey link: survey link

Provide the survey code here:

e.g. 123456

Submit

Figure 37: Amazon's Mechanical Turk Survey Link instructions for potential survey participants.

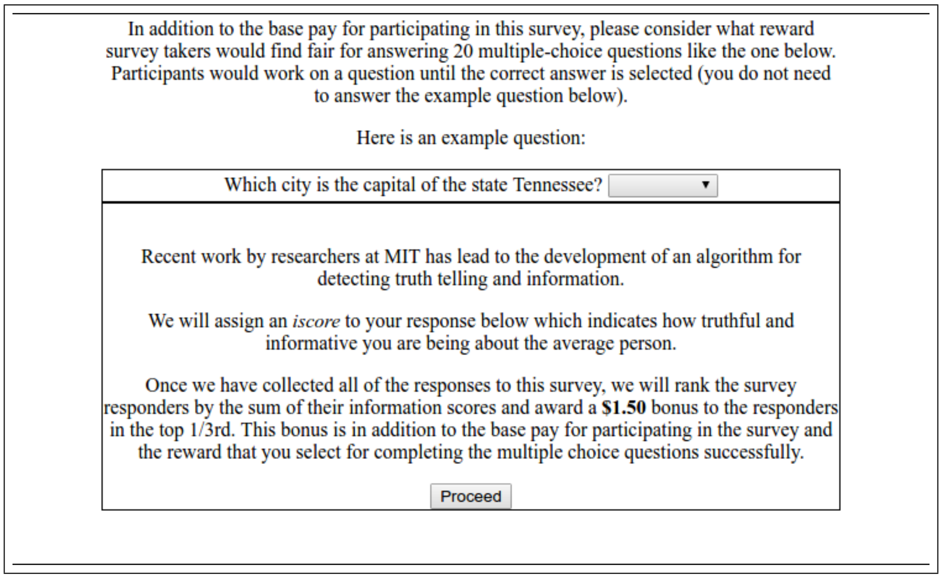

In addition to the base pay for participating in this survey, please consider what reward survey takers would find fair for answering 20 multiple-choice questions like the one below. Participants would work on a question until the correct answer is selected (you do not need to answer the example question below).

Here is an example question:

Which city is the capital of the state Tennessee?

Recent work by researchers at MIT has lead to the development of an algorithm for detecting truth telling and information.

We will assign an *iscore* to your response below which indicates how truthful and informative you are being about the average person.

Once we have collected all of the responses to this survey, we will rank the survey responders by the sum of their information scores and award a **$1.50** bonus to the responders in the top 1/3rd. This bonus is in addition to the base pay for participating in the survey and the reward that you select for completing the multiple choice questions successfully.

Proceed

Figure 38: Pricing experiment instructions. Participants in all treatments are exposed to the description of the questionnaire along with an example question from the questionnaire. Participants in BTS treatments are additionally exposed to a description of the BTS bonus.

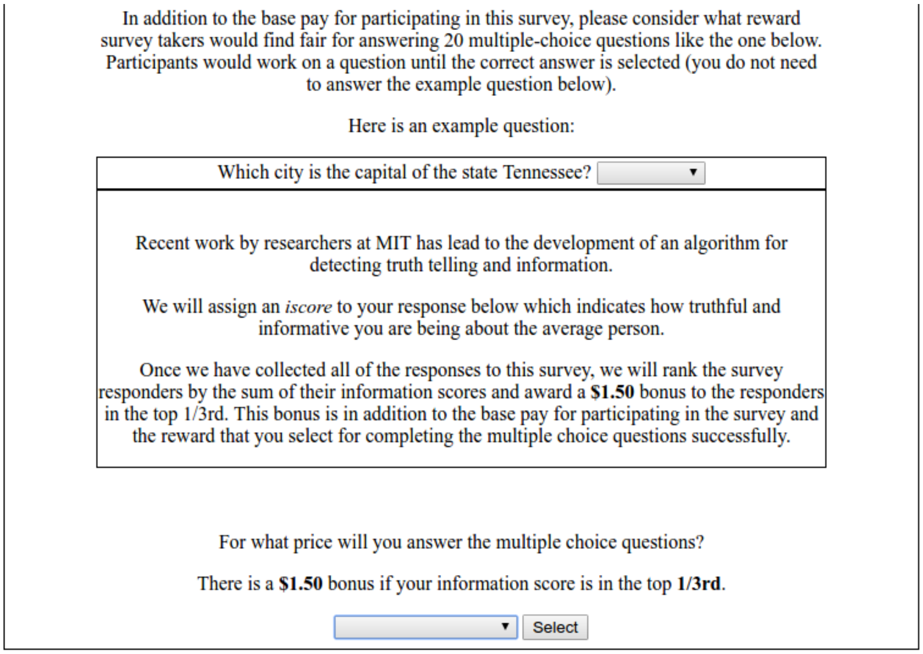

In addition to the base pay for participating in this survey, please consider what reward survey takers would find fair for answering 20 multiple-choice questions like the one below. Participants would work on a question until the correct answer is selected (you do not need to answer the example question below).

Here is an example question:

Which city is the capital of the state Tennessee?

Recent work by researchers at MIT has lead to the development of an algorithm for detecting truth telling and information.

We will assign an *iscore* to your response below which indicates how truthful and informative you are being about the average person.

Once we have collected all of the responses to this survey, we will rank the survey responders by the sum of their information scores and award a **$1.50** bonus to the responders in the top 1/3rd. This bonus is in addition to the base pay for participating in the survey and the reward that you select for completing the multiple choice questions successfully.

For what price will you answer the multiple choice questions?

There is a **$1.50** bonus if your information score is in the top **1/3rd**.

Select

Figure 39: Participants select the reward they will receive for completing the 20 multiple-choice questions in the questionnaire, or they will decline to participate. Participants in BTS treatments are reminded of the BTS bonus before selecting their reward.

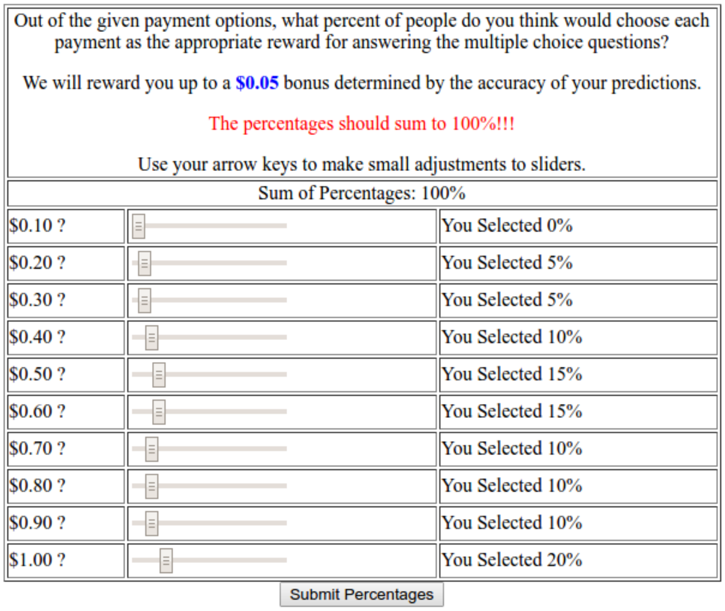

Figure 40: Participants predict the percent of all participants selecting each reward for completing the questionnaire using sliders. Note that predictions must sum to 100% for the Submit button to appear.

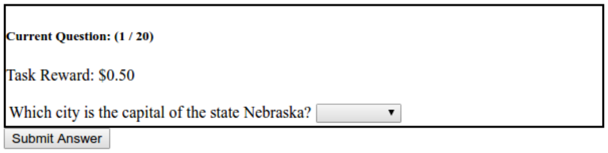

Figure 41: An example multiple-choice question from the questionnaire used in the Pricing experiment. Participants select options until the correct option is selected.

References

[1] Mason W, Watts DJ. Financial incentives and the performance of crowds. ACM SigKDD Explorations Newsletter. 2010;11(2):100–108.
